# Supplementary material for: Ethyl Lauroyl Arginate, an Inherently Multicomponent Surfactant System
Source: Molecules. 2021 Sep 29;26(19):5894. doi: 10.3390/molecules26195894 (PMC8512375; doi:10.3390/molecules26195894)
Supplement: Supplementary file 1 [file molecules-26-05894-s001.zip › molecules-1354881-supplementary.pdf]

Ethyl lauroyl arginate (LAE), an inherently multicomponent surfactant system

Agnieszka Czakaj, Ewelina Jarek, Marcel Krzan, Piotr Warszyński

Jerzy Haber Institute of Catalysis and Surface Chemistry Polish Academy of Sciences

Supporting Information

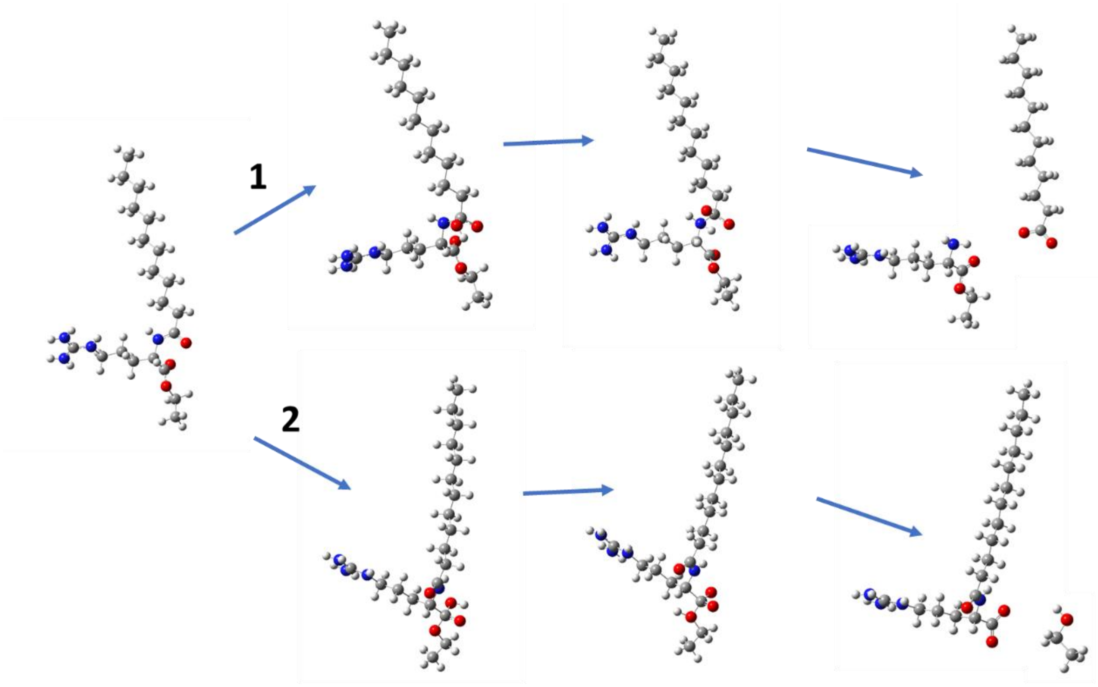

Figure S1. Scheme of base catalyzed hydrolysis of LAE. Path 1 - $LAE^{+} + OH^{-} \leftrightarrow C_{11}COO^{-} + EthylARG^{+}$; Path 2 - $LAE^{+} + OH^{-} \leftrightarrow LAS + EtOH$

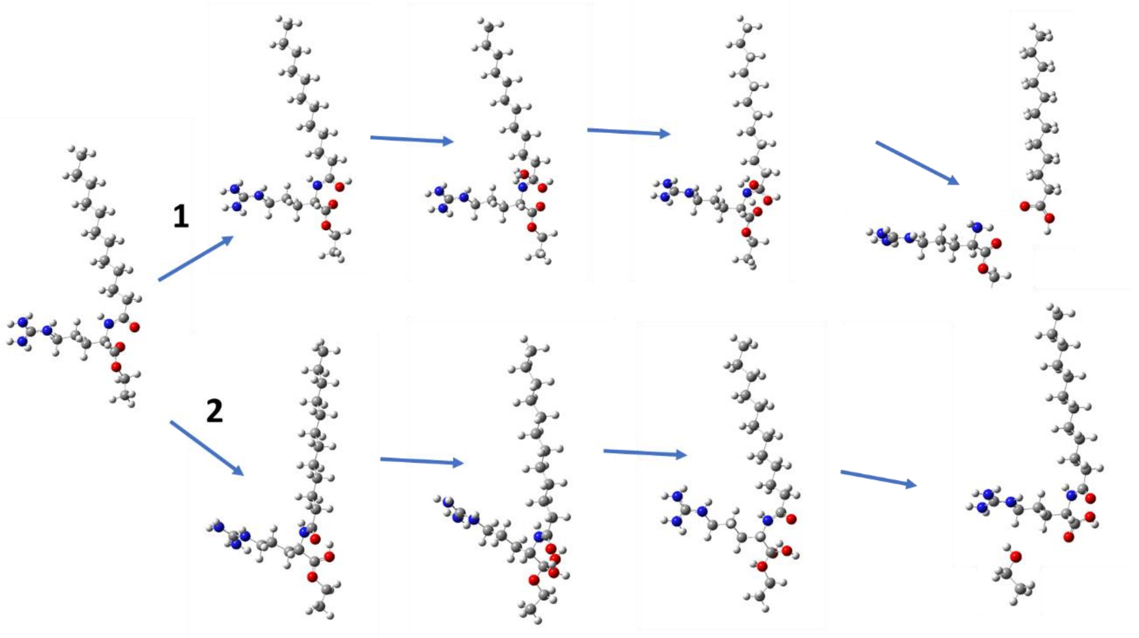

Figure S2. Scheme of acid catalyzed hydrolysis of LAE. Path 1 - $LAE^{+} + H_3O^{+} \leftrightarrow C_{11}COOH + EthylARG^{+} + H_3O^{+}$; Path 2 - $LAE^{+} + H_3O^{+} \leftrightarrow LAS^{+} + EtOH + H_3O^{+}$

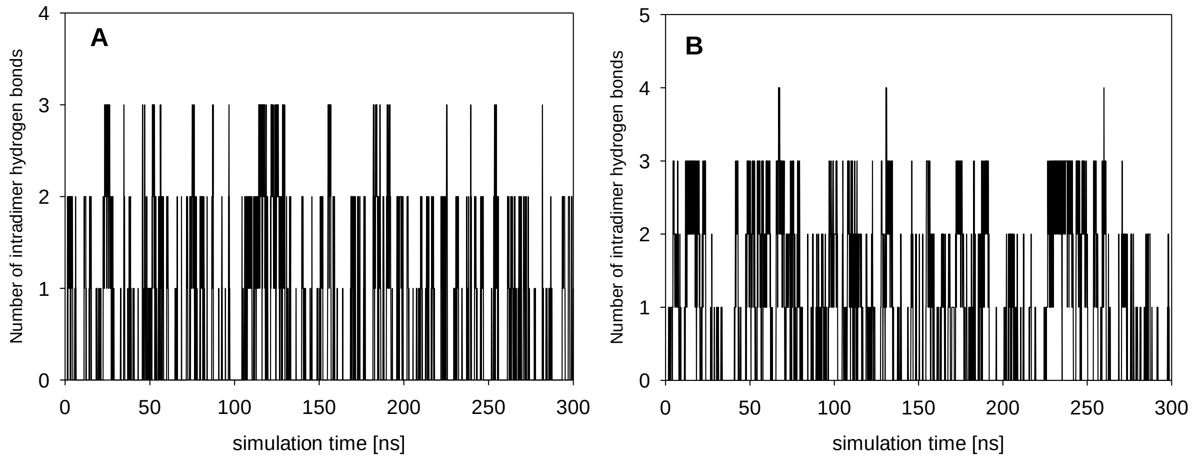

Figure S3. The variation of the number of intradimer hydrogen bonds during the simulation of the heterodimer at water/air interface. A – LAE-dodecanoate; B – LAE-LAS

Model of adsorption of surfactants and surface active hydrolysis products.

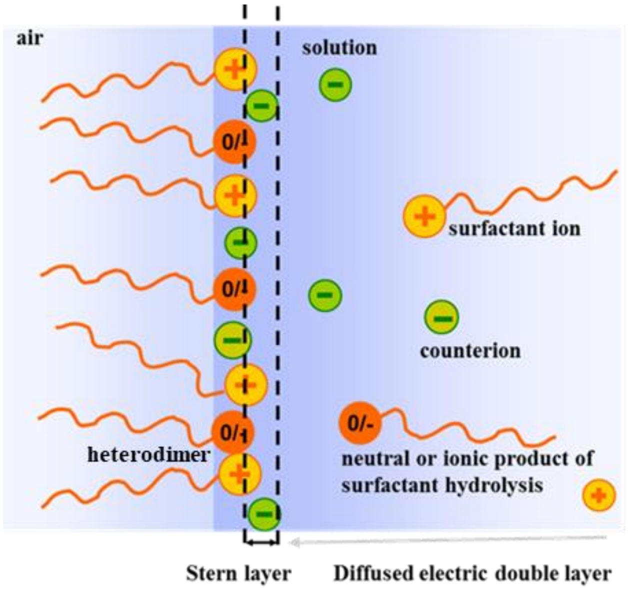

Figure S4. Schematic illustration of the main concept of the model of the adsorption of LAE and its heterodimers with hydrolysis products.

The main concept of the model is illustrated in Scheme 1. The hydrolysis of surfactants containing ester or amide bonds results in the formation of surface active products, either neutral (LAS) or negatively charged (dodecanoate anion) (see Scheme S1, where the hydrolysis products are denoted by 0/-), thus, the model of adsorption for the surfactant mixture needs to be used to describe the experimental results for the surface

tension. For that purpose we applied the extended model of adsorption of ionic/non-ionic surfactant mixtures [24,26,45] that is the extension of the surface two dimensional electrolyte (STDE) model proposed earlier by Warszyński et al. [21] to describe adsorption of ionic surfactants. We consider the system containing cationic surfactant (LAE) and anionic or neutral heterodimer with its hydrolysis product, dodecanoate anion and LAS, respectively. The system of adsorption equation derived from the equilibrium condition for the transfer from the solution to the Stern layer at the solution surface can be formulated as:

$$\frac{a_s}{\alpha_s}\exp\left(-\frac{e\psi_s}{kT}\right)\left(1-\theta_s-\sum_i\theta_{h,i}-\sum_i\theta_{a,i}\right)=\theta_s\exp\left[-2H_s\left(\theta_s+\sum_i\theta_{h,i}\right)\right]\exp\left(\frac{\phi_s}{kT}\right) \quad (1)$$

for the cationic surfactant,

$$\frac{a_{h,i}}{\alpha_{h,i}}\exp\left(-\frac{z_{h,i}e\psi_s}{kT}\right)\left(1-\theta_s-\sum_i\theta_{h,i}-\sum_i\theta_{a,i}\right)^{g_{h,i}}=\theta_{h,i}\exp\left[-2H_s\left(\theta_s+\sum_i\theta_{h,i}\right)\right]\exp\left(\frac{z_{h,i}\phi_{h,i}}{kT}\right) \quad (2)$$

for the hydrolysis products ($z_{h,i}=0$for neutral, -1 for anionic),

$$\frac{a_{a,i}}{\alpha_{a,\mathrm{i}}}\exp\left(\frac{e\psi_s}{kT}\right)\left(1-\theta_s-\sum_i\theta_{h,i}-\sum_i\theta_{a,i}\right)^{g_{a,i}}=\theta_{a,i}\exp\left(\frac{\phi_{a,i}}{kT}\right) \quad (3)$$

for the non-surface active anions of the electrolyte ($Cl^-$and OH^-).

The symbols in the above equations denote: $a_s, a_{h,i}, a_{a,i}$ - the activities of the respective components that can be calculated from the extended Debye – Hückel theory of strong electrolyte solutions - for the neutral species equal to their concentrations, $\theta_s=\Gamma_s/\Gamma_{s\infty}$ is the relative surfactant surface concentration, whereΓ_s is its surface (excess) concentration and $\Gamma_{s\infty}$is the limiting surfactant surface concentration at the maximal coverage, $\theta_{h,i}=\Gamma_{h,i}/\Gamma_{h\infty,i}, \theta_{a,i}=\Gamma_{a,i}/\Gamma_{a\infty,i}, \theta_n=\Gamma_n/\Gamma_{n\infty}, \Gamma_{h,i}, \Gamma_{a,i}, \Gamma_{H\infty}, \Gamma_{C\infty}$ and $\Gamma_{n\infty}$ are the same quantities for dimers and electrolyte anions; $g_s, g_{h,i}$, and $g_{a,i}$ are the ratios of the size of surfactant cations, dimers and electrolyte anions relatively to the size of the adsorption site ($g_s=1$ for the sake of simplicity), H_s is the surface interaction parameter accounting mainly for the attractive lateral interactions among the adsorbed surfactant hydrophobic tails,α_s is the "surface activity" of surfactant ion, being a measure of the standard free energy of adsorption after separating the contribution of the electric component, $\alpha_{h,i}$ is the same parameter for the respective heterodimers and $\alpha_{a,i}$ are the "surface activities" electrolyte anions that are a measure of their affinity to the surface layer, $\phi_s, \phi_{h,i}, \phi_{a,i}$, are the corrections for the activity of the two dimensional electrolyte in the surface layer accounting for the lateral interaction between ions. In the derivation of equations 1-3 we assumed that non-surface active cations resulting from the hydrolysis (see Scheme 1) and H_3O^+ (HCl) or Na^+ (NaOH) added to adjust pH, do not penetrate Stern layer due to

electrostatic repulsion of the positively charged interface. The electric potential of the Stern layer, ψ_S, can be found from:

$$\psi_s = \psi_d + \frac{\sigma\delta}{\varepsilon_0 \varepsilon_s} \qquad (4)$$

while the diffuse layer potential at the boundary between the Stern layer and the diffuse part of electric double layer can be determined from the formula:

$$\psi_d = \frac{2kT}{e} \sinh^{-1}\left(\frac{\sigma e}{2\varepsilon_0 \varepsilon_s kT\kappa} \right) \qquad (5)$$

where: e is the elementary charge, k is the Boltzmann constant, ε_0 is the vacuum dielectric permittivity, ε is the dielectric constant of the solution, κ is the Debye - Hückel reciprocal length

$$\sigma = F\left(\Gamma_s - \sum_i \Gamma_{h,i} - \sum_i \Gamma_{a,i} \right) \qquad (6)$$

is the surface charge density, F is the Faraday constant, δ is the thickness of the Stern layer and ε_S is the dielectric constant in the Stern layer. We assumed that adsorption of non-ionic surface active molecules does not influence the electric properties of the Stern layer.

The procedure of solving the system of Eqs. 1-6 and the detailed interpretation of parameters are described elsewhere [21,26]. By the numerical solution of this system of equations, the surface concentration of all components in the Stern layer can be determined directly. Total surface excess concentration Γ_j^T of all components has to include adsorption of all electrolyte and surfactant ions in the diffuse part of the electric double layer, where the distribution of ions has to be found using the solution of Poisson-Boltzmann equation. The surface tension of the solution can be predicted by integration of the Gibbs equation for the mixture of ionic–nonionic surfactant:

$$d\gamma = -RT\left(\sum_j \Gamma_j^T d\ln a_j\right) \qquad (7)$$

From the fit of the calculated isotherm to the experimental data the parameters of the model for investigated system can be obtained.

Table S1

Best fit parameters of STDE adsorption model

**Surfactant/Model parameter**	**LAE**	**LAE-dodecanoate anion**	**LAE-LAS**
$\Gamma_{s\infty}[mol/cm^2]$	$5.4x10^{-10}$	$7.2x10^{-10}$	$4.5x10^{-10}$
$\alpha_s[mol/dm^3]$	$5.0x10^{-6}$	$7.0x10^{-7}$	$5.0x10^{-7}$
H_s	4.5	4.5	4.5

**Other model parameters** [21]	
g_a	0.64
$\alpha_{Cl^-}[mol/dm^3]$	10000
$a_{Cl^-}\ or\ a_{OH^-}[nm]$	0.35
$\delta\ [nm]$	0.35
ε_s	24

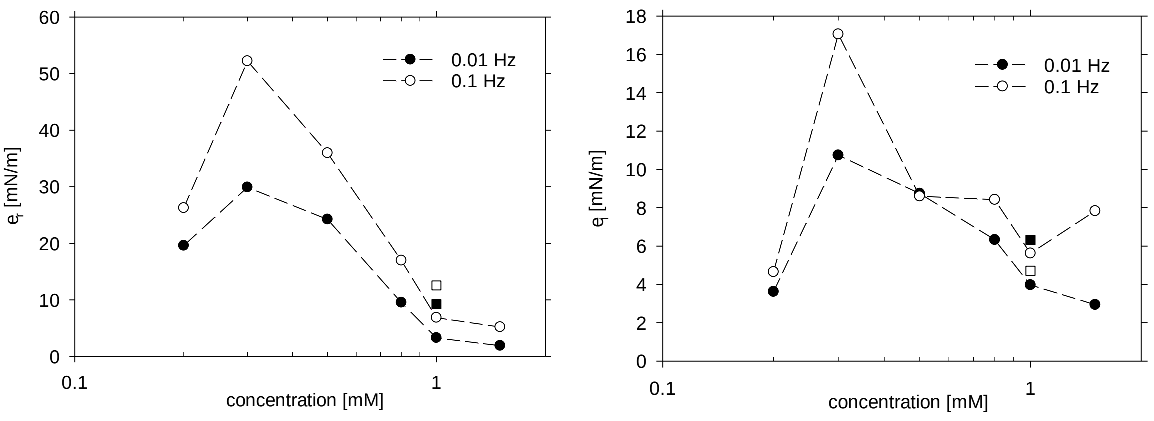

Figure S5. The dependence of the real (right) and imaginary (left) part of the dilational elastic modulus on LAE solution concentration for the drop oscillation frequency 0.01 Hz and 0.1 Hz. Squares the values for 1 mM LAE solution stored for two weeks.

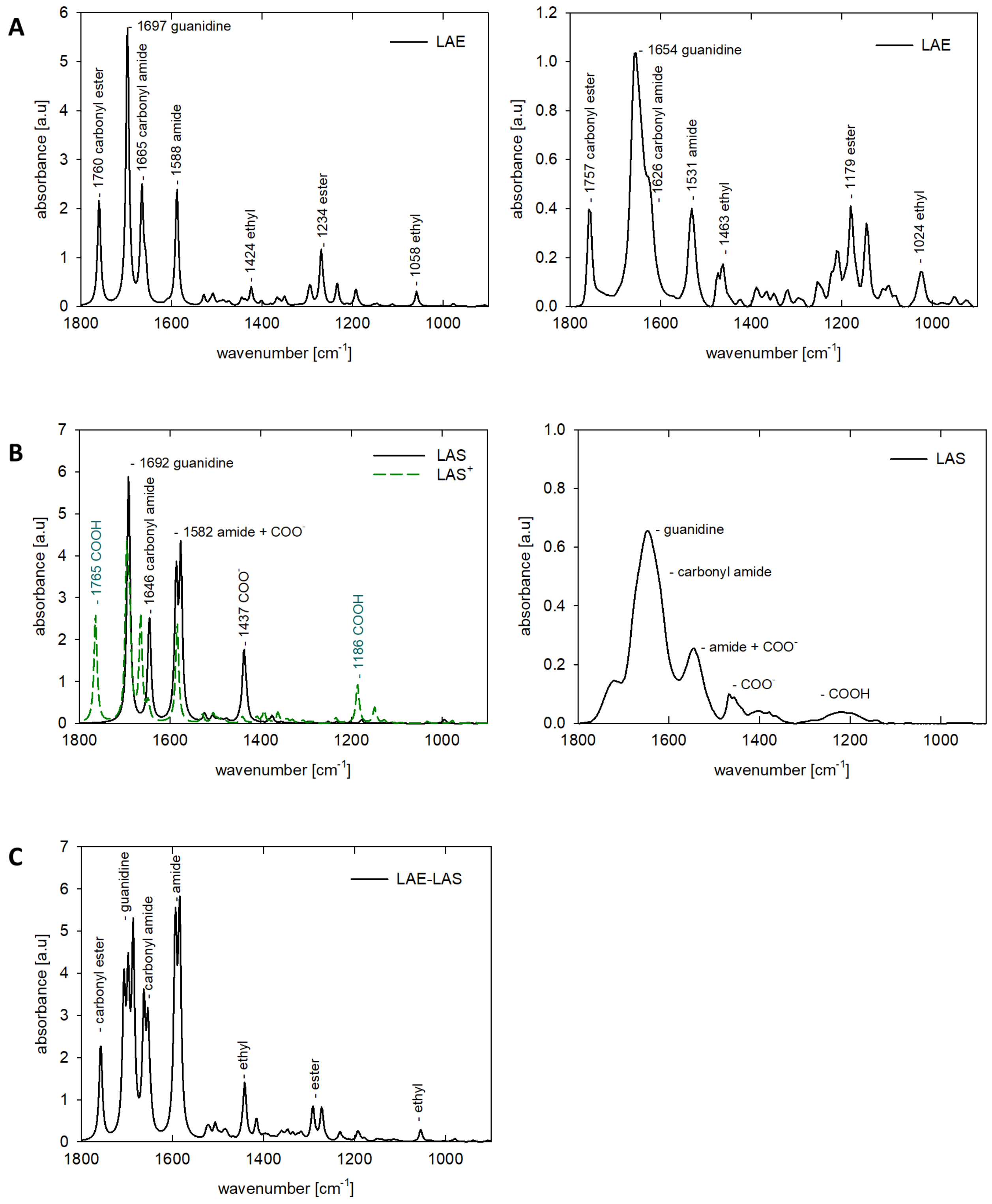

Figure S6. Infrared spectra of A – LAE, B – LAS, C – LAE-LAS heterodimer. Left – spectra resulting from the DFT computations (for LAS non-protonated and protonated), right – experimental spectra for LAE and LAS
